# Artificial ER-Mitochondrion Tethering Restores Erg6 Localization and Lipid Droplet Formation in *Hansenula polymorpha Δpex23* and *Δpex29* Cells

**DOI:** 10.1177/25152564251336908

**Published:** 2025-04-18

**Authors:** Haiqiong Chen, Rinse de Boer, David C. Lamb, Steven L. Kelly, Ida J. van der Klei

**Affiliations:** 1 Molecular Cell Biology, Groningen Biomolecular Sciences and Biotechnology Institute, University of Groningen, Groningen, The Netherlands; 2 639919 Faculty of Medicine, Health and Life Science, Swansea University, Swansea, UK

**Keywords:** Pex23, Pex29, Erg6, membrane contact sites, lipid droplet, mitochondria

## Abstract

Pex23 proteins are a family of fungal endoplasmic reticulum proteins. *Hansenula polymorpha* contains four members, two of which, Pex24 and Pex32, function in endoplasmic reticulum-peroxisome membrane contact sites. In the absence of the other two members, Pex23 and Pex29, mitochondria are fragmented and lipid droplet numbers are reduced. We here show that in *Δpex23* and *Δpex29* cells an increased portion of the lipid droplet protein Erg6 (C24-methyltransferase), an enzyme involved in ergosterol biosynthesis, localizes to mitochondria. Erg6 relocalization and the reduction in lipid droplet numbers are suppressed by an artificial endoplasmic reticulum-mitochondrion tether protein. Sterol measurements showed that the presence of Erg6 at mitochondria did not cause major changes in the overall sterol composition. Our findings suggest that Pex23 and Pex29 play a role in endoplasmic reticulum-mitochondrion contact sites which prevent mitochondrial mislocalization of Erg6.

## Introduction

Pex23 family proteins are fungal endoplasmic reticulum (ER) membrane proteins, initially discovered as peroxins, proteins important for the formation of peroxisomes (Jansen et al., 2021). Later studies revealed that some members of this family are important for lipid droplet (LD) formation or normal mitochondrial morphology and function (Wang et al., 2018; Chen et al., 2024). Pex23 family proteins accumulate at membrane contact sites (MCS), including peroxisome-ER contacts, peroxisome-LD contacts and nuclear-vacuole-junctions (NVJs, Wu et al., 2020; Chen et al., 2024). They share several structural features, including a reticulon-like domain (Joshi et al., 2016) and a C-terminal Dysferlin (DysF) domain, whose function is still unknown (Yan et al., 2008; Wu et al., 2020; Deori and Nagotu, 2022).

All yeasts contain multiple Pex23 family proteins (Jansen et al., 2021). *Saccharomyces cerevisiae* has five (Pex28, Pex29, Pex30, Pex31 and Pex32), while *Hansenula polymorpha* contains four (Pex23, Pex24, Pex29, Pex32) (Jansen et al., 2021). *S. cerevisiae* Pex28, Pex30 and Pex32 and *H. polymorpha* Pex24 and Pex32 are components of peroxisome-ER MCS (Wu et al., 2020; Ferreira and Carvalho, 2021). The absence of HpPex24 or HpPex32 results in a loss of peroxisome-ER MCSs, accompanied by major defects in peroxisome membrane expansion. These defects can be rescued by an artificial ER-peroxisome tether protein, underscoring the importance of HpPex24 and HpPex32 in peroxisome-ER MCSs (Wu et al., 2020). In addition to its role in peroxisome-ER MCSs, ScPex30 also functions in *de novo* peroxisome formation from the ER (David et al., 2013; Joshi et al., 2016; Mast et al., 2016).

The absence of *S. cerevisiae* Pex30 or *H. polymorpha* Pex23 or Pex29 results in a reduction in the number of LDs (Joshi et al., 2018; Wang et al., 2018). Moreover, in cells lacking HpPex23 or HpPex29 mitochondria are fragmented and cell growth is retarded (Chen et al., 2024). The latter phenotype, but not mitochondrial fragmentation, is suppressed upon introduction of an artificial ER-mitochondrion tether protein (designated ERMIT) (Chen et al., 2024). This observation supports a role of HpPex23 and HpPex29 in MCSs as well.

We here show that introduction of ERMIT in *H. polymorpha Δpex23* or Δ*pex29* cells also restores LD numbers. Fluorescence microscopy (FM) analysis revealed that the absence of HpPex23 or HpPex29 results in changes in the distribution of Erg6, a C24-methyltransferase involved in ergosterol synthesis (Jordá and Puig, 2020). In WT *H. polymorpha* the bulk of Erg6 localizes to LDs and the ER. Interestingly, in *Δpex23* and *Δpex29* cells Erg6 also occurs at mitochondria. Introduction of ERMIT restores both the Erg6 localization pattern and LD numbers. We propose that HpPex23 and HPex29 function at mitochondrion-ER MCSs, where they prevent mitochondrial Erg6 localization.

## Results

### The Absence of Pex23 or Pex29 Does Not Alter LD Size

**Figure 1.**
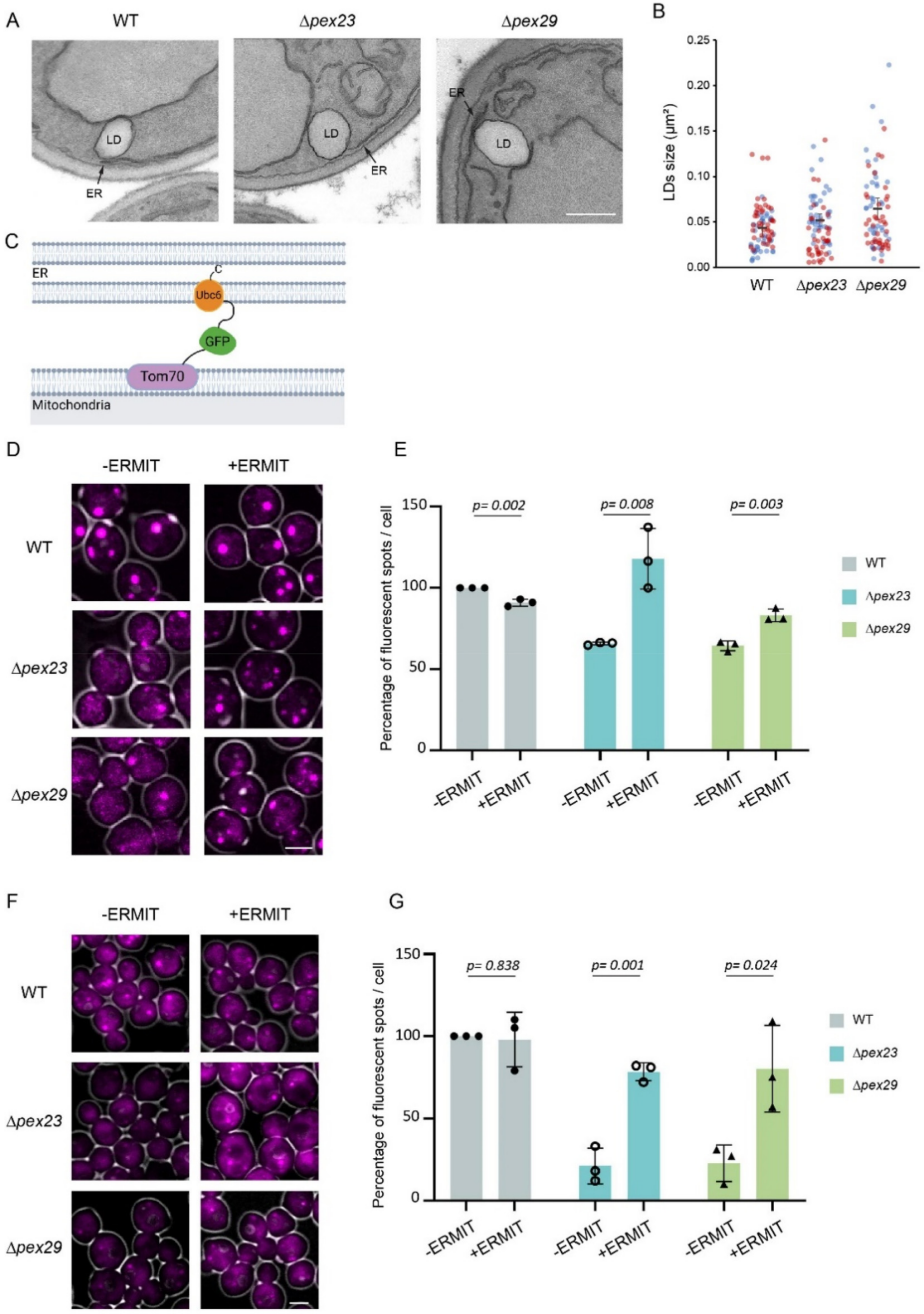
Introduction of ERMIT restores LD abundance in *Δpex23* and *Δpex29*. (A) Electron microscopy (EM) images of thin sections of KMnO_4_-fixed glucose-grown cells of the indicated strains. ER, endoplasmic reticulum; LD, lipid droplet. Scale bar: 500 nm. Representative images are shown. (B) Quantification of LD size using EM analysis of thin sections. Data are presented as mean ± SD from two independent experiments (n = 2) of 40 random LD-containing sections. (C) Schematic representation of the ERMIT tether. (D, F) CLSM Z stack images of the indicated strains producing Erg6-mKate2 (D) or stained with Nile Red (F) with or without ERMIT. The same grey scale values were used for all strains (See Figure S2 for adapted image processing). Scale bar: 2 µm. (E,G) Normalized LD abundance based on Erg6-mKate2 (E) or Nile Red marked puncta (G) observed in Z-stack CLSM images. The abundance in WT was set to 100%. Data represent the mean from three independent experiments (n = 3) with 300 cells analyzed per experiment.

Using FM analysis, we previously showed that in *H. polymorpha Δpex23* and *Δpex29* cells LD numbers are reduced [7]. We now asked whether the size of LD changed as well. To this purpose we analyzed thin sections by electron microscopy (EM), as the limited resolution of FM does not allow to accurately determine LD sizes. LDs can be readily recognized in thin sections of *H. polymorpha* WT, *Δpex23* and *Δpex29* cells, as expected (Figure 1A). Quantitative analysis of LD surface areas revealed no significant differences, indicating that the absence of Pex23 or Pex29 does not impact LD size (Figure 1B). Interestingly, the size of LDs is more variable in both mutant strains as compared to the WT control.

The decrease in LDs was not further enhanced in cells of a *Δpex23 Δpex29* double deletion strain, compared to the two single deletion strains (Figure S1).

### An Artificial Mitochondrion-ER Tether Suppresses Reduction in LDs in *Δpex23* and *Δpex29*

We previously showed that the introduction of an artificial mitochondrion-ER tether (ERMIT) restored growth of *Δpex23* and *Δpex29* cells (Chen et al., 2024). ERMIT consists of the mitochondrial membrane protein Tom70 at the N-terminus, followed by GFP and the tail anchored ER protein Ubc6 at the C-terminus (Figure 1C). We previously showed that it localizes to mitochondria and the ER, as expected (Chen et al., 2024). We now asked whether ERMIT suppressed the reduction in LD numbers. Using cells producing Erg6-mKate2 as LD marker and confocal laser scanning microscopy (CLSM), enhanced LD numbers were observed in *Δpex23* and *Δpex29* cells containing ERMIT (Figure 1D and E). A similar result was obtained when Nile Red was used as LD marker (Figure 1F and G, S2). Lower percentages of fluorescent spots were observed when Nile Red was used to mark LDs, due to differences in limit of detection compared to using Erg6-mKate2 as LD marker. Based on these observations we conclude that ERMIT suppresses the reduction in LD numbers in *Δpex23* and *Δpex29* cells.

### An Enhanced Portion of Erg6 Localizes to Mitochondria in *Δpex23* and *Δpex29* Cells

**Figure 2.**
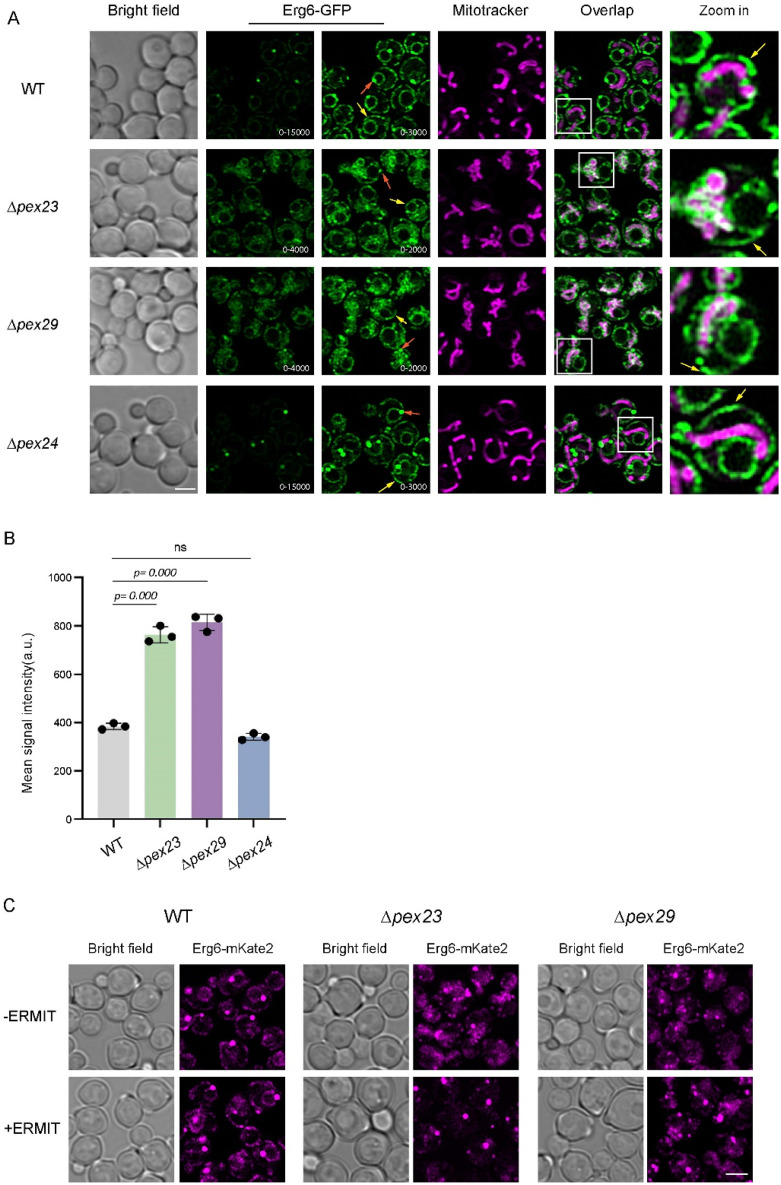
Erg6 is present at LDs, ER and mitochondria in *H. polymorpha Δpex23* and *Δpex29* cells. (A) Single plane CLSM Airyscan images showing the localization of Erg6-GFP in glucose-grown cells of the indicated strains. GFP images are shown at two different intensity values. The minimum and maximum pixel values used for the GFP channel are indicated. Mitochondria are marked with MitoTracker Red. Orange arrows indicate LDs; yellow arrows indicate the ER. White boxes in the Overlap images indicate the regions shown in the zoom-in views. Scale bar: 2 µm. (B) Quantification of Erg6-GFP mean fluorescence intensities at mitochondria, labelled with MitoTracker Red, in the indicated strains. Data are presented as mean ± SD from three independent experiments (n = 3) with 200 cells analyzed per experiment. (C) Single-plane CLSM (Airyscan) images showing Erg6-mKate2 localization in the indicated strains with or without ERMIT. Scale bar: 2 µm.

In an *S. cerevisiae dga1Δ are1Δ are2Δ* triple deletion strain defective in LD formation, Erg6 protein relocalizes from LDs to the ER (Jacquier et al., 2011). CLSM analysis revealed differences in Erg6-mKate2 fluorescence patterns in *H. polymorpha Δpex23* and *Δpex29* cells, which also show reduced LD abundance, compared to the WT control (Figure 1D). To study Erg6 localization in more detail, we changed the tag into GFP, which has a higher brightness than mKate2, and imaged the cells using Airyscan CLSM, a technique that has an approximately two-fold higher resolution and sensitivity compared to conventional CLSM analysis (Huff, 2015). Using this approach, Erg6-GFP fluorescence was detected at LDs, the ER and mitochondria (marked with MitoTracker) in *Δpex23* and *Δpex29* cells (Figure 2A). The unusual mitochondrial morphology in *Δpex23* and *Δpex29* cells is due to fragmentation and clustering as reported before (Chen et al., 2024). The additional staining at mitochondria is not due to increased ER strands associated with or enwrapping mitochondria in *Δpex23* and *Δpex29* cells, as demonstrated before by EM analysis of these cells (Chen et al., 2024). In WT and *Δpex24* control cells, in which LD numbers are unchanged (Chen et al., 2024), Erg6-GFP was predominantly present at LDs, but also detectable at the ER (Figure 2A). Quantitative analysis supported the enhanced mitochondrial Erg6-GFP signal in *Δpex23* and *Δpex29* cells (Figure 2B).

Next we asked whether ERMIT restored Erg6 distribution. To this purpose we compared cells with and without ERMIT, using Erg6-mKate2 and Airyscan CLSM imaging. We could not use GFP tagged Erg6, because ERMIT already has a GFP tag (Figure 1C; Chen et al., 2024). As expected, cells of the WT control strain exhibited clear LD spots along with fluorescently labelled ER, while *Δpex23* and *Δpex29* cells displayed additional fluorescent structures, which correspond to mitochondria as demonstrated above (Figure 2C, compare Figure 2A). Interestingly, fluorescence intensities increased at spots in cells producing ERMIT, especially in *Δpex23* cells (Figure 2C). This indicates that a larger fraction of Erg6-mKate2 occurs at LDs in *Δpex23* and *Δpex29* cells upon introduction of ERMIT. Hence, enhanced physical mitochondria-ER contacts support Erg6 localization at LDs in conjunction with LD formation in *Δpex23* and *Δpex29* cells.

### HpPex23 and HpPex29 Localize at Specialized ER Regions in the Vicinity of LDs, but are Not Essential for the Formation of LD MCSs

**Figure 3.**
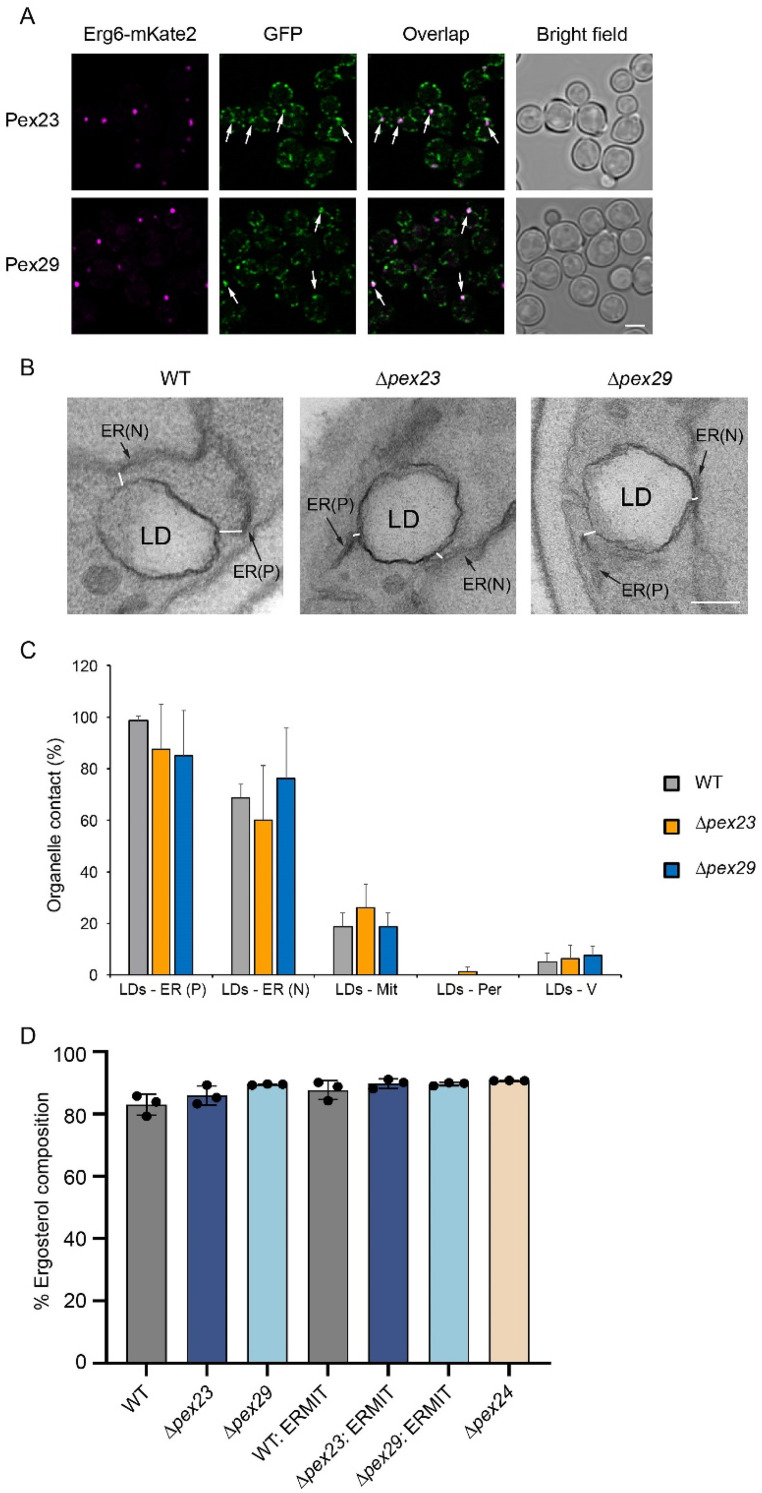
Pex23 and Pex29 accumulate at LD-MCSs, but are not essential for their formation. (A) CLSM (Airyscan, single plane) images showing the co-localization of Pex23-GFP and Pex29-GFP with Erg6-mKate2 in glucose-grown WT cells. The arrows indicate mKate spots overlapping with GFP spots. Scale bar: 2 µm. (B) Examples of EM images of thin sections of KMnO_4_-fixed glucose-grown cells of the indicated strains. White lines indicate examples of distances between two membranes, as used for the quantifications in Figure 3C. ER(N), nuclear endoplasmic reticulum; ER(P), peripheral endoplasmic reticulum; LD, lipid droplet. Scale bar: 200 nm. (C) Quantification of the distances below 30 nm between LDs and other cell organelles in the indicated strains using sections of KMnO_4_-fixed, glucose-grown cells. The percentages indicate the percentage of LDs in the five different categories. Mit, mitochondria; V, vacuole. Data are mean ± SD of two independent experiments (n = 2 using 40 random sections from each experiment). (D) The ergosterol content in the total cellular sterols of the indicated strains. Data are mean ± SD from three independent experiments. A student's t-test revealed no statistically significant differences.

*S. cerevisiae* Pex30, a member of the Pex23 family, localizes to specialized ER subdomains where LDs are formed (Choudhary and Schneiter, 2020; Ferreira and Carvalho, 2021). To study whether *H. polymorpha* Pex23 and Pex29 also accumulate at these regions*,* we performed co-localization studies using cells producing GFP-tagged Pex23 or Pex29 together with Erg6-mKate2 as an LD marker. As shown in Figure 3A, Erg6-mKate2 spots invariably localize close to regions of GFP fluorescence puncta, indicating that *H. polymorpha* Pex23-GFP and Pex29-GFP occur at specialized ER regions in the vicinity of LDs. Both proteins are also observed at other ER regions (Figure 3A). These include NVJs and ER-mitochondrion MCSs, as shown in our previous reports (Wu et al., 2020; Chen et al., 2024).

Given the importance of MCSs in LD biogenesis (Liao et al., 2022), we next studied whether the absence of Pex23 or Pex29 affects LD MCSs, by measuring the distances between the membrane using EM (See for examples Figure 3B). This analysis showed that LDs in *Δpex23* and*Δ pex29* cells exhibited similar distances to other organelles as in the WT control strain (Figure 3C). Hence, Pex23 and Pex29 are not essential for the formation of LD MCSs.

### Ergosterol Biosynthesis is Unaltered in *Δpex23* and *Δpex29* Cells

The Erg6 protein, delta (24)-sterol C-methyltransferase, is responsible for the methylation of the sterol intermediate zymosterol at C-24 to produce fecosterol in the ergosterol biosynthetic pathway. We investigated the changed distribution of Erg6 protein in *Δpex23* and *Δpex29* cells affecting the biosynthesis of ergosterol. As shown in Figure 3C, no significant differences in ergosterol levels were observed. In all strains examined, including those producing ERMIT, similar percentages of ergosterol, approximately 90% of the total sterol content, were calculated (Figure 3D). This indicates that the changed localization of a portion of the Erg6 protein does not lead to any major changes in the overall composition of sterols in the cells.

## Discussion

Here we show that *H. polymorpha* Pex23 and Pex29 are crucial for maintaining proper subcellular localization of Erg6 at LDs and the ER. Deletion of *PEX23* or *PEX29* resulted in reduced LD numbers, accompanied by the redistribution of a substantial portion of Erg6 protein to mitochondria. This redistribution was lost upon introduction of an artificial mitochondrion-ER tether (ERMIT), accompanied by the restoration of LD numbers. These data show that enhancing physical contacts between the ER and mitochondria can mitigate the disturbed cellular Erg6 distribution and a reduction in LD biogenesis caused by the absence of Pex23 or Pex29.

Our current data support a common role for Pex23 family proteins at MCSs. *H. polymorpha* Pex24 and Pex32 have been demonstrated to be crucial for ER-peroxisome MCSs (Wu et al., 2020) and HpPex23 localizes to NVJs and mitochondrion-ER MCSs (Wu et al., 2020; Chen et al., 2024). Being MCS resident proteins is furthermore underscored by the restoration of peroxisome (in *Δpex24* and *Δpex32*) or LD biogenesis defects (in *Δpex23* and *Δpex29*) by artificial tether proteins.

In *S. cerevisiae* Erg6 predominantly localizes to the ER and LDs, but Erg6 was also detected in isolated mitochondrial outer membrane vesicles by proteomic analysis (Zahedi et al., 2006). Most likely only minute amounts occur in mitochondria of WT *S. cerevisiae*, because so far ScErg6-GFP was never shown to localize to mitochondria by FM (Jacquier et al., 2011).

The sterol measurements showed that overall ergosterol biosynthesis occurs normally in *H. polymorpha Δpex23* and *Δpex29* cells, in a similar manner as shown in the WT or *Δpex24* controls. The functional status of Erg6 among altered traffic is uncertain. Apparently, sufficient Erg6 protein localizes in the ER to allow normal ergosterol biosynthesis.

**Figure 4.**
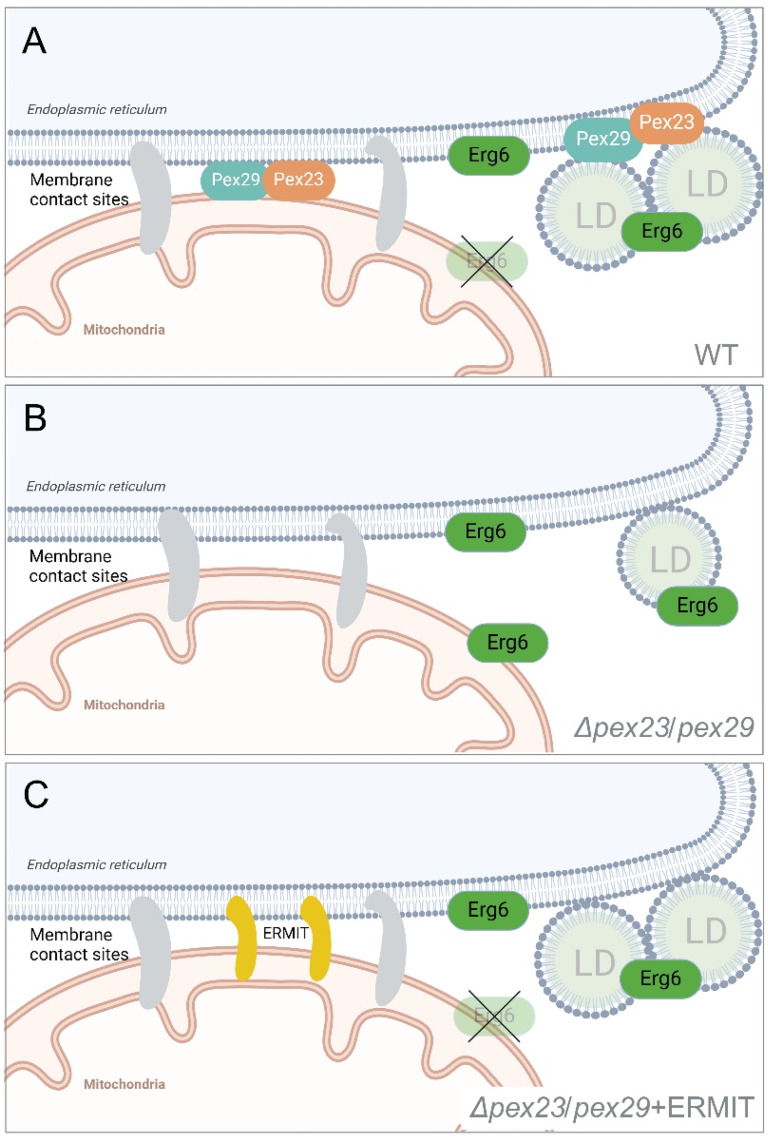
Hypothetical model of Pex23 and Pex29 protein function in LD biogenesis and Erg6 localization. (A) In WT cells, Erg6 initially sorts to the ER and localizes at LDs when these organelles are formed. Specialized ER regions involved in LD formation contain Pex23 and Pex29, but both proteins also occur at mitochondrion-ER contacts, where they either prevent transfer of Erg6 from the ER to mitochondria or facilitate the retrieval of Erg6 back to the ER from where it can be transferred to LDs. (B) In cells lacking Pex23 and Pex29 proteins, the portion of Erg6 that localizes to the mitochondria cannot be efficiently retrieved to the ER, or the trafficking of Erg6 from the ER to mitochondria is stimulated. This restricts Erg6 availability for LD formation, thereby reducing LD formation. (C) The introduction of the artificial tether ERMIT enhances the retrieval of Erg6 from the mitochondria back to the ER, supporting LD formation, or prevents transport of Erg6 from ER to mitochondria. Figure created using BioRender.

*S. cerevisiae* Erg6 initially localizes to the ER and is transferred to LDs during their biogenesis. We speculate that *H. polymorpha* Pex23 and Pex29 contribute to the correct localization of Erg6 at the ER and LDs either by preventing sorting of Erg6 to mitochondria or by stimulating transfer of mitochondrial Erg6 back to the ER (Figure 4). It is tempting to speculate that transport of Erg6 from the ER to mitochondria occurs via ER-Surf, a process that involves ER-mitochondria MCSs (Koch et al., 2024). However, other (in)direct pathways are possible as well. As an artificial ER-mitochondrion tether partially suppressed the *Δpex23* and *Δpex29* phenotypes, Pex23 and Pex29 may play a regulatory role(s) in ER-Surf. Since Erg6 is important for LD formation, a decrease in Erg6 availability at the ER and LDs may explain the reduced LD numbers in *Δpex23* and *Δpex29* (Figure 4). Notably, studies on organelle interactions in *S. cerevisiae* indicated that Erg6 physically interacts with Pex30, a sequence homolog of HpPex23 (Pu et al., 2011). This interaction may be part of the molecular mechanisms involved in regulating Erg6 distribution.

Summarizing, in the absence of *H. polymorpha* Pex23 family proteins Pex23 and Pex29 LD abundance is reduced in conjunction with enhanced localization of Erg6 to mitochondria. This defect most likely relates to their role in ER-mitochondria MCSs, because an artificial ER-mitochondrion tether suppresses the Erg6 mislocalization and LD phenotype of *Δpex23* and *Δpex29* cells.

## Materials and Methods

### Strains and Growth Conditions

*Escherichia coli* DH5α and *H. polymorpha* cells were grown described before (Van Dijken et al., 1976; Chen et al., 2024), except that 0.5% (w/v) glucose was used as carbon source in *H. polymorpha* cultures. Strains were constructed as describe before (Faber et al., 1994) (see Tables S1 and S2 for an overview of strains and plasmids used). Plasmid pHIPX-Pex14-mKate2 was constructed by digesting pHIPX PMP47-mKate2 with the restriction enzymes *Mlu*I and *Not*I, which results in a 2000 bp fragment containing the selection marker *LEU* gene. This fragment was isolated and inserted in pHIPZ Pex14-mKate2, which was digested with *Mlu*I and *Not*I. A plasmid encoding Erg6-mKate2 was generated by amplifying a PCR fragment encoding the C-terminus of *ERG6* (primers *ERG6*-fw 5′- CCCAAGCTTGAGAGAGCCAACAACTACGC-3′ and *ERG6*-rev 5′-CGCGGATCCTTTAGCATCTAATGGCTTTC-3′) using genomic DNA of wild type *H. polymorpha* as a template. The resulting PCR fragment was cut with *Hin*dII/*Bam*HI and ligated into *Hin*dII/*Bgl*II digested pHIPX-Pex14-mKate2 to generate plasmid pHIPX-Erg6-mKate2, which was transformed in *Δyku80, Δpex23, Δpex29* and *Δyku80*: MIT-ER*, Δpex23*: MIT-ER and *Δpex29*: MIT-ER. *Bsm*I-linearized pHIPZ-Pex23-mGFP and *Bgl*II-linearized pHIPZ-Pex29-mGFP were transformed into *Δyku80*: Erg6-mKate2 strain (Faber et al., 1994).

### Microscopy

Electron microscopy, staining of lipid droplets and mitochondria as well as wide field and airyscan fluorescence microscopy were performed as described previously (de Boer and van der Klei, 2023; Chen et al., 2024). The number of LDs was quantified from Z-stacks of CLSM images using a custom-made FIJI plugin for automated analysis (Thomas et al., 2015). The amount of Erg6-GFP present on mitochondria was quantified using single plane Airyscan images of MitoTracker Red stained cells in FIJI. The MitoTracker channel was auto-thresholded. For each mitochondrial structure the mean Erg6-GFP fluorescence intensity was measured (Shihan et al., 2021).

### Sterols Measurement and Data Analysis

To prepare samples for sterol extraction, cells were grown to the exponential phase in glucose medium, and 15 OD_660_ units of culture were harvested by centrifugation at 3200 rpm for 3 min. Cells were washed with sterile water three times and flash frozen in liquid nitrogen. Sterol extraction and data analysis were performed as described previously (Ahmad et al., 2019).

## Supplemental Material

sj-docx-1-ctc-10.1177_25152564251336908 - Supplemental material for Artificial ER-Mitochondrion Tethering Restores Erg6 Localization and Lipid Droplet Formation in *Hansenula polymorpha Δpex23* and *Δpex29* Cells Supplemental material, sj-docx-1-ctc-10.1177_25152564251336908 for Artificial ER-Mitochondrion Tethering Restores Erg6 Localization and Lipid Droplet Formation in *Hansenula polymorpha Δpex23* and *Δpex29* Cells by Haiqiong Chen, Rinse de Boer, David C. Lamb, Steven L. Kelly and Ida J. van der Klei in Contact

## References

[bibr1-25152564251336908] Ahmad S Joseph L Parker JE Asadzadeh M Kelly SL Meis JF Khan Z (2019). ERG6 and ERG2 are major targets conferring reduced susceptibility to amphotericin B in clinical candida glabrata isolates in Kuwait. Antimicrob Agents Chemother 63. 10.1128/AAC.01900-18. PMC6355561 30455247

[bibr2-25152564251336908] Chen H de Boer R Krikken AM Wu F van der Klei IJ (2024). Hansenula polymorpha cells lacking the ER-localized peroxins Pex23 or Pex29 show defects in mitochondrial function and morphology. Biol Open 13. 10.1242/bio.060271 PMC11139031 38682287

[bibr3-25152564251336908] Choudhary V Schneiter R (2020). Lipid droplet biogenesis from specialized ER subdomains. Microb Cell (Graz, Austria) 7, 218–221. 10.15698/MIC2020.08.727. PMC7380455 32743002

[bibr4-25152564251336908] David C Koch J Oeljeklaus S Laernsack A Melchior S Wiese S Schummer A Erdmann R Warscheid B Brocard C (2013). A combined approach of quantitative interaction proteomics and live-cell imaging reveals a regulatory role for endoplasmic reticulum (ER) reticulon homology proteins in peroxisome biogenesis. Mol Cell Proteomics 12, 2408. 10.1074/MCP.M112.017830. 23689284 PMC3769320

[bibr5-25152564251336908] de Boer R van der Klei IJ (2023). Correlative light- and electron microscopy in peroxisome research. Methods Mol Biol 2643, 93–104. 10.1007/978-1-0716-3048-8_7. 36952180

[bibr6-25152564251336908] Deori NM Nagotu S (2022). Peroxisome biogenesis and inter-organelle communication: An indispensable role for Pex11 and Pex30 family proteins in yeast. Curr Genet 68, 537–550. 10.1007/S00294-022-01254-Y. 36242632

[bibr7-25152564251336908] Faber KN Haima P Harder W Veenhuis M Ab G (1994). Highly-efficient electrotransformation of the yeast Hansenula polymorpha. Curr Genet 25, 305–310. 10.1007/BF00351482. 8082173

[bibr8-25152564251336908] Ferreira JV Carvalho P (2021). Pex30-like proteins function as adaptors at distinct ER membrane contact sites. J Cell Biol 220. 10.1083/jcb.202103176. PMC8374871 34402813

[bibr9-25152564251336908] Huff J (2015). The airyscan detector from ZEISS: Confocal imaging with improved signal-to-noise ratio and super-resolution. Nat Methods 12, i–ii. 10.1038/nmeth.f.388.

[bibr10-25152564251336908] Jacquier N Choudhary V Mari M Toulmay A Reggiori F Schneiter R (2011). Lipid droplets are functionally connected to the endoplasmic reticulum in Saccharomyces cerevisiae. J Cell Sci 124, 2424–2437. 10.1242/JCS.076836. 21693588

[bibr11-25152564251336908] Jansen RLM Santana-Molina C van den Noort M Devos DP van der Klei IJ (2021). Comparative genomics of peroxisome biogenesis proteins: Making sense of the PEX proteins. Front Cell Dev Biol 9, 654163. 10.3389/fcell.2021.654163. 34095119 PMC8172628

[bibr12-25152564251336908] Jordá T Puig S (2020). Regulation of ergosterol biosynthesis in Saccharomyces cerevisiae. Genes (Basel) 11, 1–18. 10.3390/GENES11070795. PMC7397035 32679672

[bibr13-25152564251336908] Joshi AS Huang X Choudhary V Levine TP Hu J Prinz WA (2016). A family of membrane-shaping proteins at ER subdomains regulates pre-peroxisomal vesicle biogenesis. J Cell Biol 215, 515–529. 10.1083/JCB.201602064. 27872254 PMC5119935

[bibr14-25152564251336908] Joshi AS Nebenfuehr B Choudhary V Satpute-Krishnan P Levine TP Golden A Prinz WA (2018). Lipid droplet and peroxisome biogenesis occur at the same ER subdomains. Nat Commun 9, 1–12. 10.1038/s41467-018-05277-3. 30054481 PMC6063926

[bibr15-25152564251336908] Koch C Lenhard S Räschle M Prescianotto-Baschong C Spang A Herrmann JM (2024). The ER-SURF pathway uses ER-mitochondria contact sites for protein targeting to mitochondria. EMBO Rep 25, 2071–2096. 10.1038/s44319-024-00113-w. 38565738 PMC11014988

[bibr16-25152564251336908] Liao PC Yang EJ Borgman T Boldogh IR Sing CN Swayne TC Pon LA (2022). Touch and go: Membrane contact sites between lipid droplets and other organelles. Front Cell Dev Biol 10, 852021. 10.3389/fcell.2022.852021. 35281095 PMC8908909

[bibr17-25152564251336908] Mast FD Jamakhandi A Saleem RA Dilworth DJ Rogers RS Rachubinski RA Aitchison JD (2016). Peroxins Pex30 and Pex29 dynamically associate with reticulons to regulate peroxisome biogenesis from the endoplasmic reticulum. J Biol Chem 291, 15408–15427. 10.1074/jbc.M116.728154. 27129769 PMC4957030

[bibr18-25152564251336908] Pu J Ha CW Zhang S Jung JP Huh WK Liu P (2011). Interactomic study on interaction between lipid droplets and mitochondria. Protein Cell 2, 487. 10.1007/S13238-011-1061-Y. 21748599 PMC4875178

[bibr19-25152564251336908] Shihan MH Novo SG Le Marchand SJ Wang Y Duncan MK (2021). A simple method for quantitating confocal fluorescent images. Biochem Biophys Rep 25, 100916. 10.1016/J.BBREP.2021.100916. 33553685 PMC7856428

[bibr20-25152564251336908] Thomas AS Krikken AM Van Der Klei IJ Williams CP (2015). Phosphorylation of Pex11p does not regulate peroxisomal fission in the yeast hansenula polymorpha. Sci Rep 5, 11493. 10.1038/SREP11493. 26099236 PMC4477233

[bibr21-25152564251336908] Van Dijken LP Otto R Harder W (1976). Growth of Hansenula polymorpha in a methanol-limited chemostat – physiological responses due to the involvement of methanol oxidase as a key enzyme in methanol metabolism. Arch Microbiol 111, 137–144. 10.1007/BF00446560/METRICS. 1015956

[bibr22-25152564251336908] Wang S Idrissi FZ Hermansson M Grippa A Ejsing CS Carvalho P (2018). Seipin and the membrane-shaping protein Pex30 cooperate in organelle budding from the endoplasmic reticulum. Nat Commun 9, 1–12. 10.1038/s41467-018-05278-2. 30054465 PMC6063905

[bibr23-25152564251336908] Wu F de Boer R Krikken AM Akşit A Bordin N Devos DP van der Klei IJ (2020). Pex24 and Pex32 are required to tether peroxisomes to the ER for organelle biogenesis, positioning and segregation in yeast. J Cell Sci 133. 10.1242/jcs.246983. 32665322

[bibr24-25152564251336908] Yan M Rachubinski DA Joshi S Rachubinski RA Subramani S (2008). Dysferlin domain-containing proteins, Pex30p and Pex31p, localized to two compartments, control the number and size of oleate-induced peroxisomes in pichia pastoris. Mol Biol Cell 19, 885–898. 10.1091/mbc.E07-10-1042. 18094040 PMC2262989

[bibr25-25152564251336908] Zahedi RP Sickmann A Boehm AM Winkler C Zufall N Schönfisch B Guiard B Pfanner N Meisinger C (2006). Proteomic analysis of the yeast mitochondrial outer membrane reveals accumulation of a subclass of preproteins. Mol Biol Cell 17, 1436–1450. 10.1091/mbc.e05-08-0740. 16407407 PMC1382330

