# Supplementary material for: Artificial ER-Mitochondrion Tethering Restores Erg6 Localization and Lipid Droplet Formation in Hansenula polymorpha Δpex23 and Δpex29 Cells
Source: Contact (Thousand Oaks). 2025 Apr 18;8:25152564251336908. doi: 10.1177/25152564251336908 (PMC12033454; doi:10.1177/25152564251336908)
Supplement: sj-docx-1-ctc-10.1177_25152564251336908 - Supplemental material for Artificial ER-Mitochondrion Tethering Restores Erg6 Localization and Lipid Droplet Formation in Hansenula polymorpha Δpex23 and Δpex29 Cells [file sj-docx-1-ctc-10.1177_25152564251336908.docx]

**Supplementary Figures**

**
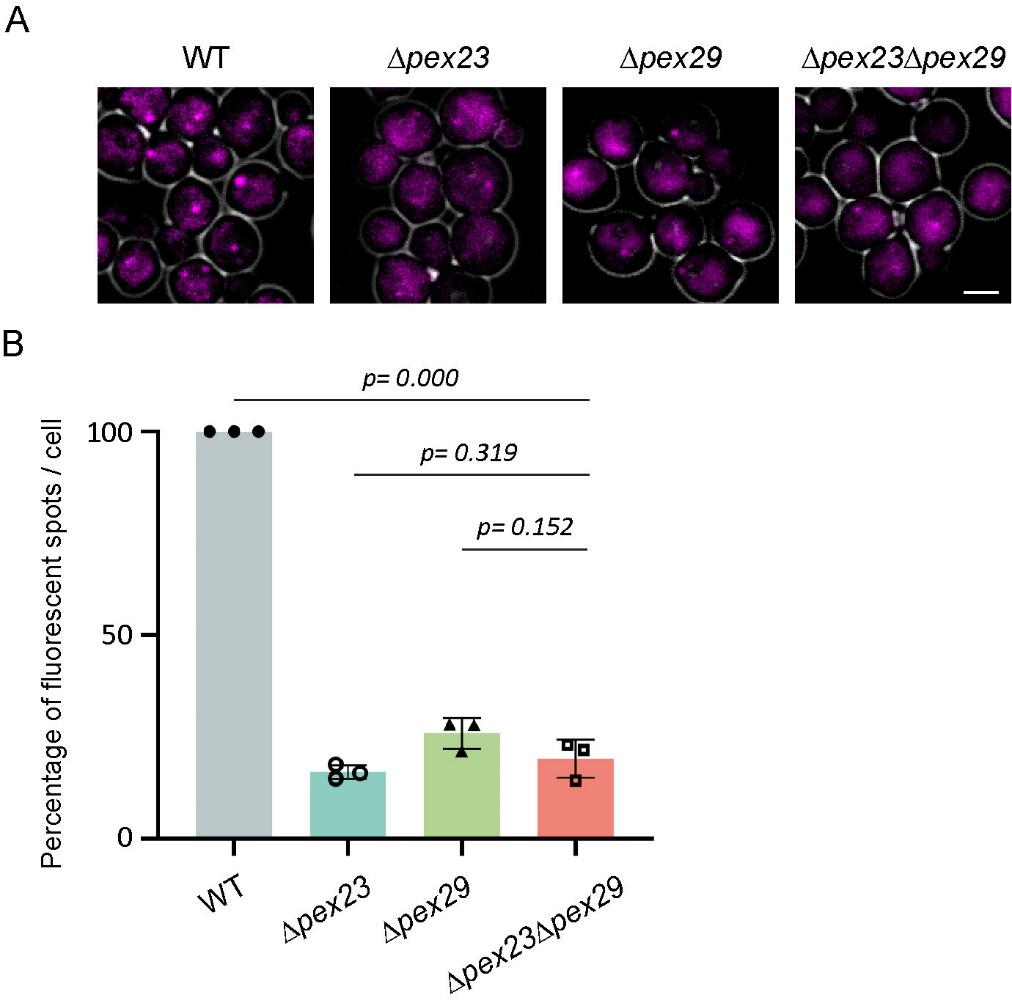
**

**Figure S1. A similar reduction in LD numbers occurs in cells of Δ*pex23*, Δ*pex29* and the Δ*pex23* Δ*pex29* double deletion strain.** (A) CLSM Z stack images of cells of the indicated strains stained with Nile Red. Scale bar: 2 µm. (B) Normalized LD abundance based on Nile Red marked puncta. The abundance in WT was set to 100 %. Data represent the mean from three independent experiments (n=3) with 300 cells analyzed per experiment.

**
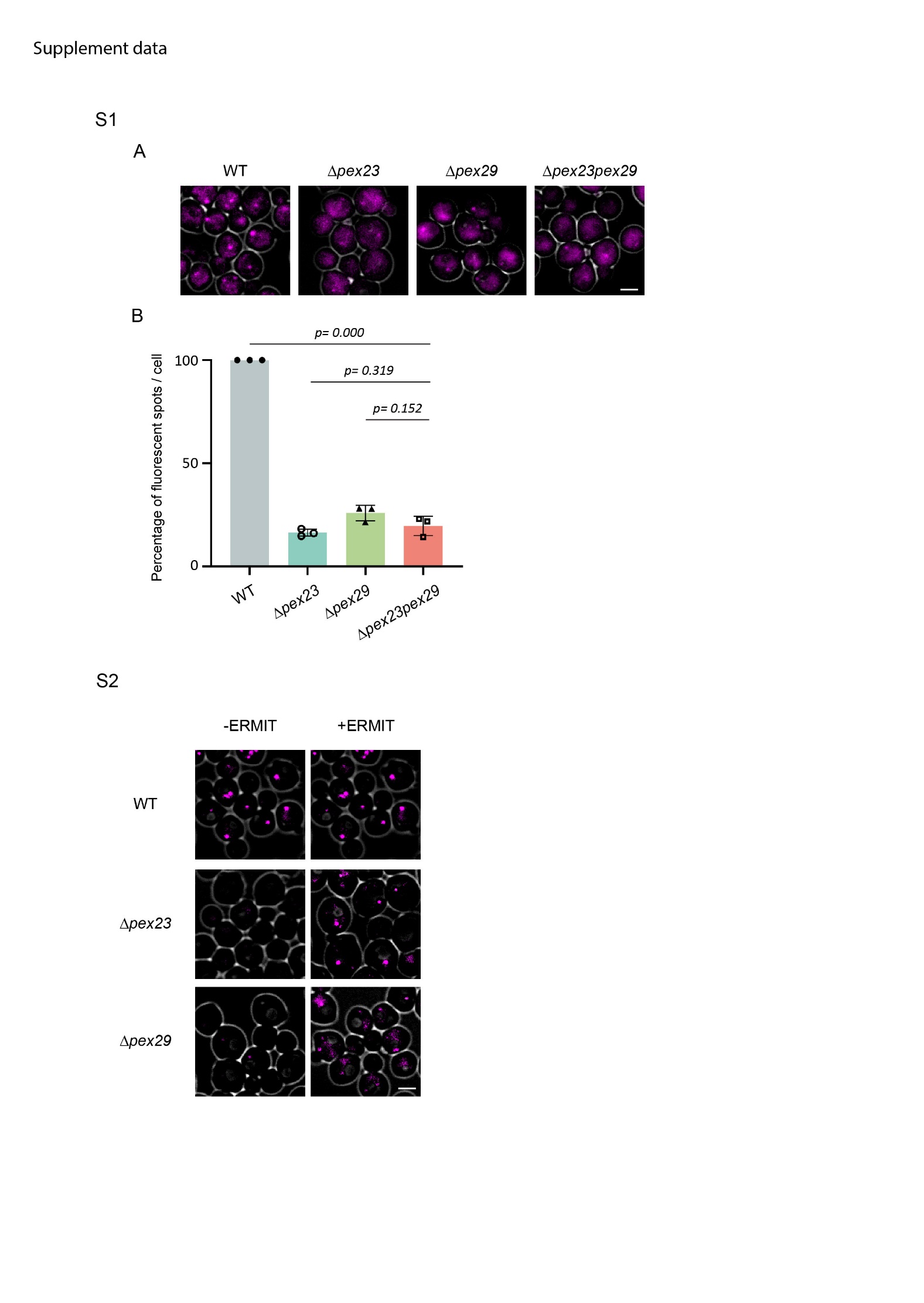
**

**Figure S2. CLSM Z stack images of cells of the indicated strains stained with Nile Red**. Image processing was not identical for all images, but optimized to visualise the Nile Red spots (belongs to Fig. 1F). Scale bar: 2 µm.

**Supplementary Tables**

**Table S1. Strains used in this study**

| **Strain** | **Characteristics** | **Reference** |
| --- | --- | --- |
| WT (*Δyku80*) | NCYC495 WT with *yku80* deletion; *leu 1.1* | (Saraya et al., 2012) |
| *Δpex23* | *Δyku80* with *PEX23* deletion; *leu 1.1,* Zeo^R^ | (Wu et al., 2020) |
| *Δpex24* | *Δyku80* with *PEX24* deletion; *leu 1.1,* Zeo^R^ | (Wu et al., 2020) |
| *Δpex29* | *Δyku80* with *PEX29* deletion; *leu 1.1*, Zeo^R^ | (Wu et al., 2020) |
| *Δpex32* | *Δyku80* with *PEX32* deletion; *leu 1.1,* Zeo^R^ | (Wu et al., 2020) |
| *Δpex23Δpex29* | *Δyku80* with *PEX23* and *PEX29* deletion; *leu 1.1,* *sh ble*, Hph^R^ | (Aksit, 2018) |
| WT: Erg6-mGFP | *Δyku80* with pHIPN-Erg6-mGFP; *leu 1.1,* Nat^R^ | (Chen et al., 2024) |
| *Δpex23*: Erg6-mGFP | *Δpex23* with pHIPN-Erg6-mGFP; *leu 1.1,* Nat^R^ | (Chen et al., 2024) |
| *Δpex24*: Erg6-mGFP | *Δpex24* with pHIPN-Erg6-mGFP; *leu 1.1,* Nat^R^ | (Chen et al., 2024) |
| *Δpex29*: Erg6-mGFP | *Δpex29* with pHIPN-Erg6-mGFP; *leu 1.1,* Nat^R^ | (Chen et al., 2024) |
| WT: Erg6-mKate2 | *Δyku80* with pHIPX-Erg6-mKate2; Zeo^R^*, LEU* | This study |
| *Δpex23*: Erg6-mKate2 | *Δpex23* with pHIPX-Erg6-mKate2; Zeo^R^*,* *LEU* | This study |
| *Δpex29*: Erg6-mKate2 | *Δpex24* with pHIPX-Erg6-mKate2; Zeo^R^*,* *LEU* | This study |
| WT: Erg6-mKate2:: Pex23-mGFP | *Δyku80* with pHIPX-Erg6-mKate2 and pHIPZ-Pex23-mGFP; *leu 1.1,* Zeo^R^*,* *LEU* | This study |
| WT: Erg6-mKate2:: Pex29-mGFP | *Δyku80* with pHIPX-Erg6-mKate2 and pHIPZ-Pex29-mGFP; *leu 1.1,* Zeo^R^*,* *LEU* | This study |
| WT: ERMIT | *Δyku80* with pHIPN18-Tom70-mGFP-Ubc6; *leu 1.1,* Nat^R^ | (Chen et al., 2024) |
| *Δpex23*: ERMIT | *Δpex23* with pHIPN18-Tom70-mGFP-Ubc6; *leu 1.1,* Zeo^R^*,* Nat^R^ | (Chen et al., 2024) |
| *Δpex29*: ERMIT | *Δpex29* with pHIPN18-Tom70-mGFP-Ubc6; *leu 1.1,* Zeo^R^*,* NAT^R^ | (Chen et al., 2024) |
| WT: ERMIT:: Erg6-mKate2 | *Δyku80* with pHIPN18-Tom70-mGFP-Ubc6 and pHIPX-Erg6-mKate2; *LEU*, Nat^R^ | This study |
| *Δpex23*: ERMIT:: Erg6-mKate2 | *Δpex23* with pHIPN18-Tom70-mGFP-Ubc6 and pHIPX-Erg6-mKate2; Zeo^R^*, LEU*, Nat^R^ | This study |
| *Δpex29*: ERMIT:: Erg6-mKate2 | *Δpex29* with pHIPN18-Tom70-mGFP-Ubc6 and pHIPX-Erg6-mKate2; Zeo^R^*, LEU*, Nat^R^ | This study |

**Table S2. Plasmids used in this study**

| **Plasmid** | **Description** | **Reference** |
| --- | --- | --- |
| pHIPZ Pex14 mKate2 | pHIPZ plasmid containing the C-terminal part of *PEX14* fused to mKate2; Amp^R^, Zeo^R^ | (Chen et al., 2018) |
| pHIPX PMP47-mKate2 | pHIPX plasmid containing the C-terminal part of *PMP47* fused to mKate2; Amp^R^, *LEU* | (Krikken et al., 2020) |
| pHIPX-Pex14-mKate2 | pHIPX plasmid containing the C-terminal part of *PEX14* fused to mKate2; Amp^R^, *LEU* | This study |
| pHIPX-Erg6-mKate2 | pHIPX plasmid containing the C-terminal part of *ERG6* fused to mKate2; Amp^R^, *LEU* | This study |

**Supplementary References:**

Aksit, A (2018). Peroxisomal membrane contact sites in the yeast *Hansenula polymorpha*. PhD Thesis, University of Groningen. research.rug.nl/en/publications/peroxisomal-membrane-contact-sites-in-the-yeast-hansenula-polymor

Chen H, Boer R de, Krikken AM, Wu F, Klei I van der (2024). *Hansenula polymorpha* cells lacking the ER-localized peroxins Pex23 or Pex29 show defects in mitochondrial function and morphology. *Biol Open* *13* doi: 10.1242/bio.060271

Chen X, Devarajan S, Danda N, Williams C (2018). Insights into the Role of the Peroxisomal Ubiquitination Machinery in Pex13p Degradation in the Yeast *Hansenula polymorph*a. *J Mol Biol* *430*, 1545–1558. doi: 10.1016/J.JMB.2018.03.033

Krikken AM, Wu H, de Boer R, Devos DP, Levine TP, van der Klei IJ (2020). Peroxisome retention involves Inp1-dependent peroxisome–plasma membrane contact sites in yeast. *J Cell Biol* *219*, e201906023. doi: 10.1083/JCB.201906023

Saraya R, Krikken AM, Kiel JAKW, Baerends RJS, Veenhuis M, van der Klei IJ (2012). Novel genetic tools for *Hansenula polymorpha*. *FEMS Yeast Res* *12*, 271–278. doi: 10.1111/J.1567-1364.2011.00772.X

Wu F, de Boer R, Krikken AM, Akşit A, Bordin N, Devos DP, van der Klei IJ (2020). Pex24 and Pex32 are required to tether peroxisomes to the ER for organelle biogenesis, positioning and segregation in yeast. *J Cell Sci* *133* doi: 10.1242/jcs.246983
